# Histone deacetylase 1 interacts with HIV-1 Integrase and modulates viral replication

**DOI:** 10.1186/s12985-019-1249-y

**Published:** 2019-11-19

**Authors:** Fadila Larguet, Clément Caté, Benoit Barbeau, Eric Rassart, Elsy Edouard

**Affiliations:** Département des sciences biologiques, and Centre de recherche BioMed, Université du Québec à Montréal, Montréal, QC Canada

**Keywords:** HIV-1, Integrase, HDAC1, Preintegration step

## Abstract

**Background:**

HIV-1 hijacks the cellular machinery for its own replication through protein-protein interactions between viral and host cell factors. One strategy against HIV-1 infection is thus to target these key protein complexes. As the integration of reverse transcribed viral cDNA into a host cell chromosome is an essential step in the HIV-1 life cycle, catalyzed by the viral integrase and other important host factors, we aimed at identifying new integrase binding partners through a novel approach.

**Methods:**

A LTR-derived biotinylated DNA fragment complexed with the integrase on magnetic beads was incubated with extracts from integrase-expressing 293 T cells. Liquid chromatography-mass spectrometry/mass spectrometry and co-immunoprecipitation/pull-down experiments were used for the identification of binding partners. Transfections of histone deacetylase 1 (HDAC1) expression vectors and/or specific siRNA were conducted in HeLa-CD4 and 293 T cells followed by infection with fully infectious NL4–3 and luciferase-expressing pseudotyped viruses or by proviral DNA transfection. Fully infectious and pseudotyped viruses produced from HDAC1-silenced 293 T cells were tested for their infectivity toward HeLa-CD4 cells, T cell lines and primary CD4+ T cells. Late RT species and integrated viral DNA were quantified by qPCR and infectivity was measured by luciferase activity and p24 ELISA assay. Results were analyzed by the Student’s *t*-test.

**Results:**

Using our integrase-LTR bait approach, we successfully identified new potential integrase-binding partners, including HDAC1. We further confirmed that HDAC1 interacted with the HIV-1 integrase in co-immunoprecipitation and pull-down experiments. HDAC1 knockdown in infected HeLa cells was shown to interfere with an early preintegration step of the HIV-1 replication cycle, which possibly involves reverse transcription. We also observed that, while HDAC1 overexpression inhibited HIV-1 expression after integration, HDAC1 knockdown had no effect on this step. In virus producer cells, HDAC1 knockdown had a limited impact on virus infectivity in either cell lines or primary CD4+ T cells.

**Conclusions:**

Our results show that HDAC1 interacts with the HIV-1 integrase and affects virus replication before and after integration. Overall, HDAC1 appears to facilitate HIV-1 replication with a major effect on a preintegration step, which likely occurs at the reverse transcription step.

## Introduction

Following entry into the target cell, the human immunodeficiency virus type 1 (HIV-1) genomic RNA is reverse transcribed into double-stranded linear DNA, which integrates into the host cell chromosome. Integration is an essential step in the HIV-1 life cycle and is catalyzed by the viral integrase (IN) present in the virion. Moreover, studies have suggested that HIV-1 IN is involved in several different steps of viral replication such as uncoating, reverse transcription, nuclear import of viral cDNA and virion maturation [1–9].

The integration reaction proceeds in three steps. The first step (3′-processing) takes place in the cytoplasm and leads to the removal of the dinucleotide from each 3’end of the viral long terminal repeat (LTR) adjacent to a conserved CA dinucleotide, thereby generating two CA-3′-hydroxyl DNA ends. In the strand transfer step, which occurs in the nucleus, CA-3′-hydroxyl DNA ends are covalently joined to 5′-phosphates in the host DNA to produce the integration intermediate. The integration is completed by gap filling of unrepaired 5′-ends of the viral DNA by cellular enzymes. Former studies have further shown that purified IN exhibits both 3′-processing and strand transfer activity in vitro. However, reactions with recombinant IN mainly resulted in the insertion of a single viral DNA end in a single strand of the duplex target DNA (for a review, see [10, 11]).

In infected cells, IN functions in the context of the preintegration complex (PIC), a large nucleoprotein complex formed in the cytoplasm with components derived from the core of the infecting virion (viral cDNA, matrix, nucleocapsid, reverse transcriptase, Vpr, and IN) and several cellular factors. A variety of cellular proteins were identified as important partners required for successful viral DNA integration in infected cells. Sorin and co-workers had previously reported that histone deacetylase I (HDAC1) could be such a partner, being incorporated in HIV-1 virions in an integrase-dependent manner as part of the Sin3a-HDAC1 complex [12]. Subsequently, Allouche and colleagues showed that HDAC1 was incorporated in a complex formed by KAP1, a protein belonging to the antiviral TRIM family, which binds IN and induces its deacetylation [13]. Interestingly, many studies had been devoted to elucidate the role of HDAC proteins in HIV-1 silencing in latently infected cells [14–22]. Class I histone deacetylase inhibitor, such as vorinostat, can disrupt latency in HIV-1-infected patients on antiretroviral therapy [23, 24]. However, few studies have examined the role of HDAC proteins in HIV-1 replication at steps other than the well-characterized regulation of the proviral expression [12, 13].

The identification and characterization of new IN cellular cofactors remains an important goal as it provides a more complete understanding of viral integration and can potentially lead to the development of new antiviral drugs. We have recently developed an in vitro purification protocol relying on the use of streptavidin-coupled magnetic beads and a portion of the viral U3 LTR region as bait for integrase-containing complex followed by mass spectrometry analysis. In this report, we confirm that HDAC1 interacts with HIV-1 IN. We demonstrate that HDAC1 exerts a control on early and late events of HIV-1 replication. In early events, HDAC1 is involved in a preintegration step, possibly during reverse transcription. In later steps, HDAC1 appears to inhibit gene expression from the integrated viral DNA. We also show that HDAC1 depletion in virion producer cells has a limited effect on virion infectivity toward Jurkat cells and human primary CD4+ T cells. Altogether, our results highlight a complex role for HDAC1 in HIV-1 replication.

## Materials and methods

### Plasmids

The FLAG-HDAC1 expression vector was constructed as follows. The cDNA coding sequence of human HDAC1 flanked by EcoRI restriction sites was generated by PCR amplification from a cDNA clone (ATCC, MGC-8378) with the following primers: sense 5′-GGGAATTCATGGCGCAGACGCAGGGC-3′ and antisense 5′-CGGAATTCTCAGGCCAACTTGACCTC-3′. The PCR product was digested by EcoRI and inserted into the pBact-FLAG vector (derived from pBact-myc [25]) to generate p-FLAG-HDAC1. To construct a 6xHis.HDAC1 expression vector, the HDAC1 cDNA was subcloned from p-FLAG-HDAC1 into the EcoRI site of pTrcHis (B). HIV-1 proviral DNA clones pNL4–3 and pNL4–3.Luc.R-E- and the VSV-G envelope expression vector were obtained from the NIH AIDS Research & Reference Reagent Program (Germantown MD) [26–28]. The plasmid pcDNA3.1(+)-LEDGF-HA was kindly provided by Dr. Thibault Mesplède (McGill University, Montréal, Canada), while the pCEP-IN^s^ala-Flag vector was a generous gift from Dr. Zeger Debyser [29].

### Antibodies

Rabbit HIV-1 HXB2 integrase antisera (obtained from Dr. Duane P. Grandgenett) [30] and anti-HIV-1 integrase monoclonal antibodies were provided by the NIH AIDS Research & Reference Reagent Program (cat. #757 and #758; 8G4 cat. # 7375 and 2C11 cat. #7374). The monoclonal anti-DDX5 antibody (C10) was a generous gift from Dr. Hans Stahl (Universität des Saarlands, Sarrebruck, Germany). The following antibodies were purchased from Sigma-Aldrich (St-Louis MO): rabbit polyclonal anti-Flag, mouse monoclonal anti-Flag-M2 and mouse monoclonal β-Actin antibodies. Monoclonal anti-DDX17 antibodies were obtained from Santa Cruz Biotechnology Inc. (Dallas TX), while monoclonal anti-HDAC1 antibodies were purchased from Cell signaling Technology (Danvers MA). All secondary conjugated antibodies were provided by GE Health Care (Chicago, IL).

### siRNA synthesis

All siRNAs used in this study were purchased from Qiagen (Mississauga, Canada). Hs_HDAC1_5 (Cat. no SI02634149) and Hs_HDAC1_6 (Cat. no SI02663472) target the 3′-UTR and the coding sequence of the HDAC1 mRNA, respectively. The GFP-22 siRNA (Cat. no 1022064) was used as a negative control. siRNA transfection was performed using the HiPerfect transfection reagent (Qiagen) according to manufacturer’s instructions.

### Cell culture

Human embryonic kidney 293 T cells were cultured in Dulbecco’s Modified Eagle Medium (DMEM) containing 10% fetal bovine serum (FBS), 2 mM L-glutamine, 50 U/ml penicillin and 50 μg/ml streptomycin (DMEM-complete). 293 T-IN^s^ala cells, stably expressing high levels of HIV-1 integrase, were obtained from Dr. Zeger Debyser [31] and grown in DMEM-complete in the presence of 200 μg/ml hygromycin B. HeLa-CD4-LTR-β-gal (HeLa-CD4) cells were obtained from Dr. Michael Emerman [32] through the NIH AIDS Research & Reference Reagent Program and were maintained in DMEM supplemented with 10% FBS, 200 μg/ml G418 and 100 μg/ml hygromycin B. Jurkat T cells were cultured in RPMI-1640 medium containing 10% FBS and 2 mM L-glutamine and antibiotics. LuSIV cells were obtained through the NIH AIDS Research & Reference Reagent Program from Drs. Jason W. Roos and Janice E. Clements [33]. LuSIV cells were grown in RPMI medium containing 10% FBS, 2 mM sodium pyruvate, 10 mM HEPES, 2 mM L-glutamine, 50 U/ml penicillin, 50 μg/ml streptomycin and 300 μg/ml hygromycin B. This cell line was derived from CEMx174 cells stably transfected with a construct containing SIV LTR-driven luciferase reporter gene and is sensitive to infection by HIV strains, leading to Tat-mediated expression of luciferase.

### Purification of human primary CD4+ T cells

Peripheral blood samples were obtained after written consent from the donor. Following isolation of peripheral blood mononuclear cells (PBMCs) by Ficoll-Hypaque density gradient centrifugation, CD4^+^ T cells were isolated by negative selection with the human CD4^+^ T Cell Enrichment Kit (Stem Cell Technologies Inc., Vancouver, Canada). Purity of the CD4+ cell population was higher than 93% as measured by flow cytometry. Cells were cultured at a density of 2 × 10^6^ cells per ml in RPMI-1640 medium supplemented with 10% FBS, L-glutamine (2 mM) and antibiotics (50 U/ml penicillin, 50 μg/ml streptomycin and 100 μg/ml primocin). For CD4^+^ T cell activation, PHA-L (1 μg/ml) (Sigma-Aldrich) and recombinant human IL-2 (3000 U/ml) (Feldan-bio, Montreal, Canada) were added to the culture medium for 24 h prior to virus infection.

### Virus stocks

Wild-type HIV-1 virions were produced by transfection of 293 T cells with the infectious molecular clone, pNL4–3. Pseudotyped HIV-Luc virions were generated by co-transfecting the pNL4–3.Luc.R-E- construct and a VSV-G envelope expression vector using the calcium phosphate transfection protocol [34]. A semi-confluent culture of 293 T cells (previously transfected or not with siRNAs) was transfected with 20 μg of pNL4–3 plasmid or with 20 μg of pNL4–3.Luc.R-E- and 5 μg VSV-G plasmids in 10 ml DMEM-complete. Cells were washed 24 h post-transfection and cultured in fresh medium. Virion-containing supernatants were harvested 48 h after transfection, centrifuged for 5 min at 700 x g, cleared by filtration through 0.22 μm pore-size filters, aliquoted and stored at − 80 °C. Virus stocks were assayed for virion content by using an in-house double-Ab sandwich ELISA specific for the viral p24^gag^ protein [35]. Recombinant HIV-1 p24 was purchased from Feldan-bio. Anti-p24 antibodies used for the p24 ELISA were a generous gift from Dr. Michel Tremblay (Centre de Recherche du Centre Hospitalier Universitaire de l’Université Laval, Quebec City, Canada). Before each infection experiment, the virus inoculum was treated with DNase I (1 μg/ml) in the presence of MgCl_2_ (1 mM) for 30 min at 37 °C to remove contaminating DNA.

### Purification of HIV-1 integrase-containing complexes and identification of interacting proteins

Biotinylated DNA baits corresponding to identical segments of the U3 region of MuLV (316 bp) or HIV-1 LTR (313 bp) were first generated by PCR amplification from corresponding proviral DNA. Binding of either biotinylated DNA to the HIV-1 integrase was first verified by supershift assay before starting the purification. For the purification of the complex, 10 mg of streptavidin magnetic beads (Dynabeads® M-280 Streptavidin, Invitrogen) were used. The beads were washed in 1 M NaCl buffer (5 mM Tris-HCl pH 7.5, 0.5 mM EDTA, 1 M NaCl) and then incubated with 2 μg of the biotinylated DNA bait for 30 min at room temperature with rotation. Following extensive washes in 1 M NaCl buffer, beads were incubated in a binding buffer (20 mM HEPES pH 7.9, 50 mM KCl, 1 mM MgCl_2_, 0.05 mM EDTA, 5% glycerol and 1 mg/ml BSA) in the presence of 0.5 mg of nuclear extracts [36] from 293 T-IN^s^ala vs. 293 T control cells for the MuLV-derived bait. The HIV-1 LTR bait-bead complex was similarly incubated with nuclear extracts from 293 T cells transiently transfected with 4 μg of pCEP-IN^s^ala-Flag and/or 4 μg pcDNA3.1(+)-LEDGF-HA using polyethylenimine (PEI) (Polysciences, Warrington, PA) at a 7:1 ratio of PEI/total DNA in FBS-free DMEM. The mixture was incubated for 30 min at room temperature with rotation. The supernatant was discarded and following extensive washes with 0.1 M NaCl buffer, proteins were eluted in 0.5 M NaCl buffer (or glycine buffer pH 5.0).

### Mass spectrometry analyses

Protein complexes from two independent purification experiments using the MuLV LTR bait were run in parallel on 4–12% SDS-PAGE gels followed by silver staining. The presence of integrase in the fraction purified from 293 T-IN^s^ala extract was verified by Western blot using a mixture of HIV-1 IN 8G4 and 2C11 monoclonal antibodies [30]. A total of 12,293 T-IN^s^ala-specific protein bands were excised, in-gel trypsin digested and subjected to liquid chromatography-mass spectrometry/mass spectrometry at Genome Quebec Innovation Centre. The resulting dataset was blasted against NCBI databases (NCBInr 20060525 and NCBInr 20060921 for human and viral sequences, respectively).

### Co-immunoprecipitation experiments

293 T-IN^s^ala cells were seeded (1 × 10^6^ cells per 60 mm plate) and transfected with 4 μg p-FLAG-HDAC1 plasmid using the PolyFect reagent (Qiagen). Cells were washed 48 h after transfection and lysed with cold modified RIPA buffer (50 mM Tris-HCl pH 7.4, 150 mM NaCl, 1% NP40, 0.25% sodium deoxycholate, 1 mM EDTA, 1 mM sodium orthovanadate_,_ 1 mM NaF) containing complete protease inhibitors (Roche) followed by centrifugation at 10,000 x g for 15 min to pellet cellular debris. Total cell lysates (400 μg proteins) were incubated at 4 °C for 1 h with the following antibodies: anti-HIV-1 integrase, anti-FLAG or normal rabbit IgG. Protein G-sepharose beads (Invitrogen) were then added and incubation was continued overnight at 4 °C with rotation. Beads were washed four times with modified RIPA buffer, and eluted by heating at 95 °C for 5 min in 50 μl 2X electrophoresis sample buffer. Immunoprecipitation eluates were resolved by 10% SDS-PAGE. Western blot analysis was carried out as previously described [37] using a mixture of HIV-1 IN 8G4 and 2C11 monoclonal antibodies and the anti-FLAG-M2 antibody.

### Purification of recombinant 6xHis-tagged HDAC1 and pull-down assay

Recombinant 6xHis.HDAC1 protein was expressed from the pTrcHis vector in *E. coli* (DH10B). Expression was induced with 1 mM IPTG for 4 h at 37 °C. Bacteria were pelleted by centrifugation at 10,000 x g for 10 min and lysed in a ratio of 1 to 2 g (wet weight)/5 ml of lysis buffer (50 mM NaH_2_PO_4_ pH 7.4, 0.5% Tween 20, 0.5% Triton X-100, 0.5% NP40, 1% SDS, 1 mg/ml lysozyme and 1 μg/ml benzonase) during 30 min on ice. After sonication, the lysates were cleared by centrifugation at 16,000 x g for 20 min at 4 °C. Pre-cleared lysates were incubated with the Ni-NTA His.Bond® superflow resin (Novagen) equilibrated in Ni-NTA buffer [50 mM NaH_2_PO_4_ buffer pH 8.0, 150 mM NaCl and complete protease inhibitor (Roche)] in the presence of 30 mM imidazole during 2 h at 4 °C. The beads were then loaded into a column and washed with 10 column volumes of the same buffer. 6xHis-tagged proteins were eluted with 250 mM imidazole in Ni-NTA buffer. Eluted fractions were analyzed by 10% SDS-PAGE and Coomassie blue staining, then pooled and dialyzed against 20 mM HEPES pH 7.5, 1 mM DTT, 1 mM EDTA, 0.5 M NaCl and 10% glycerol. Ni-NTA His.Bond® superflow resin (50 μl) was incubated with 5 μg of purified 6xHis.HDAC1 and 1 μg of purified recombinant HIV-1_NL4–3_ IN obtained from Dr. Robert Craigie [38] (NIH AIDS Research & Reference Reagent Program) for 2 h at 4 °C in Ni-NTA buffer containing 30 mM imidazole. After 4 washes, bound proteins were eluted with 250 mM imidazole in Ni-NTA buffer, resolved by 10% SDS-PAGE and analyzed by Western blot.

### Infection and transfection experiments

HeLa-CD4 cells (8 × 10^4^) were seeded in 24-well plates and transfected with 15–20 nM siRNAs or 375 ng of expression vectors. Infection was initiated at 48 h post-transfection with fully infectious NL4–3 (10 ng p24/well) or pseudotyped HIV-Luc virions (30 ng p24 per 10^5^ cells) in infection medium (DMEM, 10% FBS, 100 μg/ml G418, 50 μg/ml hygromycin B) containing 8 μg/ml polybrene. The medium was removed 3 h after infection and cells were washed and cultured in fresh medium. For NL4–3 infection, cell-free supernatants were harvested at different time points and quantified for virus production by p24 ELISA assay. For infection with pseudotyped viruses, luciferase activities were measured from cell lysates 48 h after infection.

### Transient knockdown/overexpression and post-integration analysis

HeLa-CD4 or 293 T cells were plated in 24-well plates (1 × 10^5^ cells/well) and transfected with 20 nM siRNAs or 375 ng pFLAG-HDAC1 using the HiPerfect transfection reagent. Forty eight hours after transfection, cells were transfected with 1.6 μg of pNL4–3.Luc.R-E- in 500 μl DMEM-complete. Luciferase activities were measured in cell lysates at 24 h after transfection.

### Quantitative RT-PCR

Total RNA was isolated from siRNA-transfected cells at different time points with the TRIzol Reagent (Invitrogen). RNA samples (2.5 to 5 μg) were subjected to reverse transcription (RT) using the Omniscript reverse transcriptase kit (Qiagen) and amounts of HDAC1 and β-actin (internal control) were subsequently quantified by real-time PCR, using the SYBR Green Master Mix (Takara Bio, Inc.). PCR was conducted with 1.25–2.5 ng of cDNA along with primers specific for HDAC1 (0.5 μM) or β-actin (0.25 μM). The primer pairs were: HDAC1-sense 5′-TCCGAGACGGGATTGATGACG-3′ and HDAC1-antisense 5′-CCCAGCATCAGCATAGGCAGG-3′, β-actin sense 5′-GGGTCAGAAGGATTCCTATG-3′ and β-actin antisense 5′-GGTCTCAAACATGATCTGGG-3′. The cycling conditions were: initial denaturation at 95 °C for 10 s followed by 50 cycles of amplification (denaturation at 95 °C for 5 s, hybridization at 60–65 °C for 20 s and elongation at 72 °C for 15 s). Data were analyzed with the Real Quant Software (Roche Applied Science). Endogenous HDAC1 protein levels in siRNA-transfected cells were analyzed by Western blot.

### Quantification of HIV-1 DNA species

DNA was isolated from infected cells at different time points following the onset of infection (6, 12, 48 h and 6 days), using the FlexiGene DNA kit (Qiagen). HIV-1 DNA species were analyzed by quantitative PCR using the SYBR Green Master Mix, as described by Suzuki et al. [39] with slight modifications. Integrated viral DNA was amplified by nested Alu PCR. The first PCR round was performed on 10 ng of DNA using an Alu-sequence-specific sense primer (5′- TCCCAGCTACTCGGGAGGCTGAGG- 3′) and the HIV-1-specific antisense primer M661 (5′-CCTGCGTCGAGAGATCTCCTCTG-3′). The second PCR was performed on a 100 to 500-fold dilution of the first round PCR amplicon with sense M667 (5′-GGCTAACTAGGGAACCCACTGC-3′) and antisense AA55 (5′-CTGCTAGAGATTTTCCACACTGAC-3′) primers. The abundance of late RT products was quantified using sense primer LG564 (5′-CCGTCTGTTGTGTGACTCTGGT-3′) and antisense primer LG699 (5′-GAGTCCTGCGTCGAGAGATCT-3′) through PCR analysis of 10 ng cellular DNA. All PCR conditions included an initial denaturation at 95 °C for 10 min followed by 50 cycles of amplification (denaturation at 95 °C for 15 s and amplification at 60 °C for 1 min). For each amplification, human β-globin DNA was used as an internal control and quantified with primers 5′-CCCTTGGACCCAGAGGTTCT-3′ and 5′-CTCACTCAGTGTGGCAAAGGTG-3′. Each sample was run in triplicate. Negative PCR controls (no DNA added to the PCR mixture) were tested for each experiment. DNA extracted from non-infected HeLa-CD4 cells was included in each experiment and used to subtract background amplification. Viral DNA content of samples obtained by qPCR analysis were calculated as the percentage of the control (cells transfected with a control siRNA).

### MTS/PMS assay

siRNA-transfected cells were first washed and incubated with 400 μl of fresh medium for 1 h at 37 °C followed by the addition of a combined solution of Tetrazolium salt [3-(4,5-dimethylthiazol-2-yl)-5-(3-carboxymethoxyphenyl)-2-(4-sulfophenyl)-2H-tetrazolium] (MTS, Promega) and phenazine methosulfate (PMS, Sigma) at a final concentration of 333 μg/ml and 25 μM, respectively. After 1 h of incubation at 37 °C, absorbance was recorded at a wavelength of 490 nm.

### Luciferase assay

Transfected cells were lysed 24 h after transfection, while infected cells were lysed at 48 h post-infection. Cells were resuspended in lysis buffer [25 mM Tris-H_3_PO_4_ pH 7.8, 2 mM DTT, 1% (v/v) Triton X-100, 10% (v/v) glycerol], incubated for 30 min at room temperature with shaking and subjected to one freeze-thaw cycle. To 20 μl lysate, 100 μl of luciferase buffer [20 mM Tricine, 1.07 mM (MgCO_3_)_4_·Mg(OH)_2_·5H_2_O, 2.67 mM MgSO_4_, 0.1 mM EDTA, 270 μM coenzyme A, 470 μM D-luciferin, 530 μM ATP, and 33.3 mM DTT] were added [40]. Luciferase activities were measured as relative light units (RLU) with a Dynex MLX microplate luminometer (Dynex Technologies) and normalized to protein content of the cell lysates, as quantified with the Bradford reagent (BioRad).

### Statistical analysis

Statistical analysis was performed with Prism Software (GraphPad) using the Student’s *t*-test. Results were considered significant when *p* < 0.05. The *p-*value was calculated with reference to the control data.

## Results

### HDAC1 is identified as an integrase interacting cellular protein

**Fig. 1:**
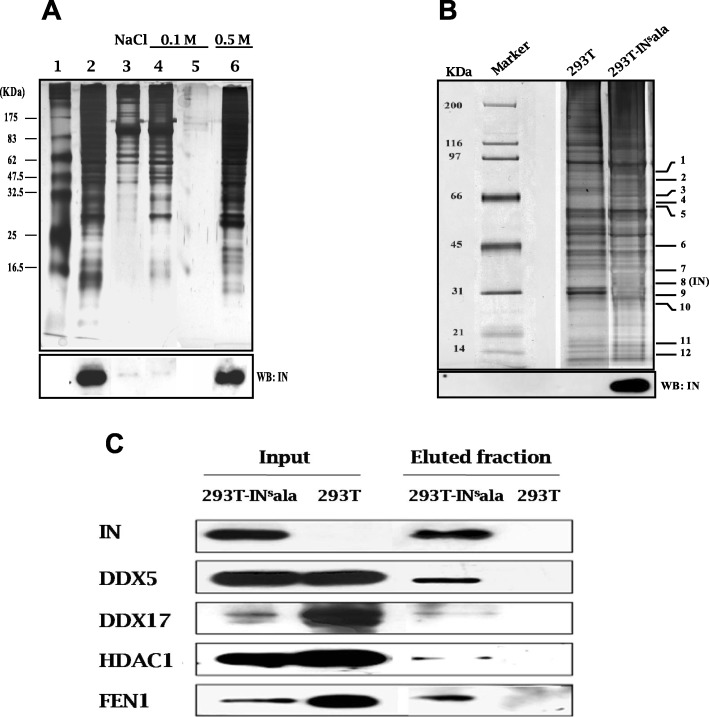
Identification of HIV-1 IN-associated proteins. A biotinylated DNA fragment corresponding to a part of the U3-LTR of CasBrE MuLV was immobilized on streptavidin-coupled magnetic beads. Nuclear extracts from stably HIV-1 IN-expressing 293 T cells (293 T-IN^s^ala) were incubated with beads. **a** After washing with 0.1 M NaCl buffer, complexes were eluted with 0.5 M NaCl buffer and resolved on a 10% SDS-PAGE. Proteins were visualized by silver nitrate staining. IN was detected by immunoblotting with anti-IN antibodies (lower panel). Marker (lane 1); 293-IN^s^ala extract (lane 2); flow-through from beads incubated with extracts (lane 3); 0.1 M NaCl washes (lane 4–5); elution (0.5 M NaCl) (lane 6). **b** Eluted samples from 293 T-IN^s^ala and 293 T cells (negative control) were migrated on SDS-PAGE (4–12%) and visualized by silver staining. Signals showing difference in profile between 293 T-IN^s^ala and control 293 T fractions are numbered (indicated on the right side of the gel) and were analyzed by mass spectrometry. Detection of IN by immunoblotting is presented in the lower panel. **c** The presence of IN and putative interacting proteins in the purified fractions from (**b**) along with extracts (input) from corresponding cell lines were analyzed by Western blot using specific antibodies (anti-FEN1, anti-DDX5, anti-DDX17, anti-HDAC1)

To purify cellular proteins in a complex with the HIV integrase, we have developed a purification method based on the use of streptavidin magnetic beads coupled to the integrase and the viral DNA of interest. Briefly, knowing that HIV-1 integrase binds the U5-U3 junction of the HIV-1 LTR and several MuLV LTR [41], two different baits containing the integrase attachment site were generated: the first one corresponds to a 316 bp region of the MuLV U3 LTR and the second one, to the equivalent 313 bp region of the HIV -1 LTR. Both DNA when complexed to beads were able to specifically bind to the HIV-1 integrase when incubated with extracts from the stably integrase-expressing 293 T-IN^s^ala cell line or from 293 T cells transiently transfected with an integrase expression vector (Fig. 1a; Additional file 1: Figure S1A). Additional experiments with the HIV-1 bait-integrase complex revealed the expected detection of the lens epithelium-derived growth factor/transcription co-activator p75 (LEDGF/p75) in HA-tagged LEDGF-expressing 293 T cells (Additional file 1: Figure S1B).

Since the MuLV LTR bait provided us with stable interaction with the integrase, protein complexes from nuclear extracts of 293 T-IN^s^ala cells (or 293 T cells, as a negative control) bound to this bait were further purified and resolved by SDS-PAGE gel followed by silver staining and Western blot analyses (Fig. 1b). Protein bands giving a different profile between 293 T-IN^s^ala and control 293 T cell fractions were excised, trypsin digested and used for identification by liquid chromatography-tandem mass spectrometry (*LC-MS/MS)*. As expected, the HIV-1 integrase was recovered from this analysis as a band near 32 kDa with 7 matching peptides (corresponding to 4 unique peptides) (Fig. 1b). These results were further confirmed by Western blot analysis. Moreover, several cellular proteins were identified, among which were the well characterized integrase cofactors: flap structure-specific endonuclease 1 (FEN1), ATP-dependent helicase II/Ku80 (XRCC5), HMG-Box proteins (HMGB-1, HMGB-2), and poly (ADP-ribose) polymerase 1 (PARP1) (Table 1). Other known integrase interacting proteins, such as LEDGF/p75, INI1 (SMARCB1), emerin (EMD) and heat shock protein 60 (HSP60) were also identified by our method (data not shown). Interestingly, using our method, we identified HDAC1 with a relatively high score. As different HDAC proteins exhibit sequence homologies, we aligned the sequences of the peptide set identified by mass spectrometry with Class I HDACs and found a 100% match with HDAC1 only, suggesting that HDAC1 was specifically co-purified with the HIV-1 integrase.

**Table 1:** HIV-1 integrase interacting proteins identified by mass spectrometry

Identified protein	Gene name	Accession no	MW (KDa)	Total score	peptide no	% coverage	Main biological function
Band 1							
RNA helicase II/Gu protein	DDX21	gi|11,890,755	80.210	658	21	27	rRNA processing
Transducin beta-like 3	TBL3	gi|16,307,379	90.347	605	16	25	Signalling, rRNA processing
DEAD (Asp-Glu-Ala-Asp) box polypeptide 1	DDX1	gi|31,565,475	83.349	462	12	20	Transcription, mRNA processing
ATP-dependent DNA helicase II (Ku80)	XRCC5	gi|10,863,945	83.222	461	11	18	DNA repair, Transcription
Transcription factor NF-AT 90 K chain – human	ILF3	gi|1,082,856	73.977	302	9	17	Transcription
Band 2							
RNA helicase II/Gu protein	DDX21	gi|11,890,755	80.210	423	13	nd	rRNA processing
HnRNP R protein	HNRNPR	gi|12,655,185	71.456	413	9	nd	mRNA splicing, processing
Structure specific recognition protein 1	SSRP1	gi|4,507,241	81.367	298	5	10	DNA repair, Transcription
Metastasis-associated protein	MTA1	gi|2,498,589	81.422	163	4	nd	Signal transduction
Band 3							
DEAD (Asp-Glu-Ala-Asp) box polypeptide 5	DDX5	gi|4,758,138	69.618	999	43	36	mRNA processing
Heat shock protein (HSP70–1/2)	HSPA1A	gi|386,785	70.110	571	12	20	Stress response
Fused in sarcoma	FUS	gi|48,145,611	53.622	525	22	23	Nuclear mRNA splicing
DEAD (Asp-Glu-Ala-Asp) box polypeptide 17	DDX17	gi|47,678,395	73.138	464	18	16	RNA processing
HNop56	NOP56	gi|2,230,878	67.206	451	10	23	rRNA processing
Band 4							
DEAD (Asp-Glu-Ala-Asp) box polypeptide 5	DDX5	gi|4,758,138	69.618	758	21	nd	mRNA processing
Paraspeckle component 1	PSPC1	gi|57,209,129	58.820	719	25	28	Transcription regulation
HnRNP R protein	HNRNPR	gi|12,655,185	71.456	423	19	16	mRNA splicing, processing
HnRNP L	HNRNPL	gi|11,527,777	64.617	404	10	23	Nuclear mRNA splicing
DEAD (Asp-Glu-Ala-Asp) box polypeptide 17	DDX17	gi|47,678,395	73.138	310	9	nd	RNA processing
Band 5							
Unnamed protein product (homologue to hnRNP L)	HNRNPL	gi|32,356	60.719	812	32	45	Nuclear mRNA splicing
Poly(ADP-ribose) polymerase	PARP1	gi|190,167	113.810	327	7	8	DNA repair, Transcription
Histone deacetylase 1	HDAC1	gi|13,128,860	55.638	319	9	22	Transcription
Telomeric repeat binding factor 2	TERF2	gi|5,032,169	55.688	302	6	20	Cell cycle
Transcription factor LSF	TFCP2	gi|476,099	57.733	244	5	nd	Transcription
Band 6							
NF45	ILF2	gi|532,313	44.898	469	11	18	Transcription
Flap structure-specific endonuclease 1	FEN1	gi|54,695,918	42.908	453	13	31	DNA repair
Vaccinia related kinase 1	VRK1	gi|4,507,903	45.790	439	12	nd	Cell cycle
DEAD (Asp-Glu-Ala-Asp) box polypeptide 48	EIF4A3	gi|13,177,790	47.126	422	10	27	mRNA metabolism, rRNA processing
HnRNP D	HNRNPD	gi|870,743	30.523	394	12	29	Transcription
Band 7							
Apurinic endonuclease	APEX1	gi|178,743	35.959	488	14	40	DNA repair, Transcription
Protein phosphatase 1, catalytic subunit, alpha isoform 1	PPP1CA	gi|4,506,003	38.229	465	9	nd	Cell cycle, Carbohydrate metabolism
protein phosphatase-1 gamma 1	PPP1CC	gi|484,316	37.271	456	9	29	Cell cycle, Carbohydrate metabolism
ELAV-like 1	ELAVL1	gi|38,201,714	36.240	393	10	30	mRNA stabilization
B23 nucleophosmin (280 AA)	NPM1	gi|825,671	31.090	332	13	21	Ribosome assembly, signal transduction, intracellular protein transport
Band 8							
Ribosomal protein L7a	RPL7A	gi|49,522,232	30.148	421	8	nd	60S ribosomal constituent
N-methylpurine-DNA glycosylase; MPG	MPG	gi|233,968	30.005	392	9	33	DNA repair
Histone H1	HIST1H1D	gi|22,770,675	22.336	323	6	nd	Nucleosome structure
Homolog of Yeast RRP4	EXOSC2	gi|12,653,909	32.996	312	6	nd	rRNA processing
Ribosomal protein S2	RPS2	gi|23,491,733	25.874	227	5	19	40S ribosomal constituent
Band 9							
High-mobility group box 2	HMGB2	gi|54,696,428	24.061	389	20	44	DNA repair and recombination
Ribosomal protein L14	RPL14	gi|1,620,022	23.902	252	6	25	60S ribosomal constituent
Ribosomal protein L13	RPL13	gi|15,431,297	24.304	225	5	27	60S ribosomal constituent
High mobility group protein B1	HMGB1	gi|48,145,843	25.035	214	13	nd	DNA repair and recombination, transcription.
Ras-related nuclear protein	RAN	gi|48,734,884	24.609	176	5	22	Cell cycle, protein transport
Band 10							
HMGB3 protein	HMGB3	gi|47,124,341	23.137	555	35	48	DNA recombination
Cleavage and polyadenylation specific factor 5	NUDT21	gi|12,655,103	26.268	466	13	30	mRNA processing
Splicing factor, arginine/serine-rich 9	SRSF9	gi|4,506,903	25.640	386	13	nd	mRNA splicing, processing
HnRNP A1 (Helix-destabilizing protein)	HNRNPA1	gi|47,939,618	34.273	352	20	nd	mRNA splicing, processing and transport
Protein C2f	EMG1	gi|2,276,396	26.474	236	4	nd	Ribosome biogenesis
Band 11							
RNA polymerase II subunit	POLR2H	gi|1,017,823	17.203	198	7	27	Transcription
Ribosomal protein S19	RPS19	gi|12,652,563	16.051	188	4	nd	40S ribosomal constituent
Histone H1b	HIST1H1B	gi|356,168	21.721	158	3	nd	Nucleosome assembly
HIST1H2BN protein	HIST1H2BN	gi|68,532,407	13.928	156	9	26	Nucleosome assembly
Band 12							
Histone H1	HIST1H1D	gi|22,770,675	22.336	323	6	nd	Nucleosome structure
HIST1H4I protein	HIST1H4I	gi|45,767,731	11.332	249	8	40	Nucleosome assembly
Histone H4	HIST4H4	gi|223,582	11.230	235	8	39	Nucleosome
HIST1H2BN protein	HIST1H2BN	gi|68,532,407	13.928	98	4	nd	Nucleosome

We further validated the presence of HDAC1 as well as three other newly identified interacting partners by Western blot analyses of purified fractions from 293T-IN^s^ala cells (Fig. 1c). All proteins were indeed detected following purification in the presence of the HIV integrase only.

These data hence demonstrated that our method allowed us to detect several known integrase binding partners, as well as new unidentified partners, such as HDAC1. As HDAC1 presented a strong reproducible signal, we focussed on this integrase binding partner.

### Direct interaction of the HIV-1 integrase with HDAC1

**Fig. 2:**
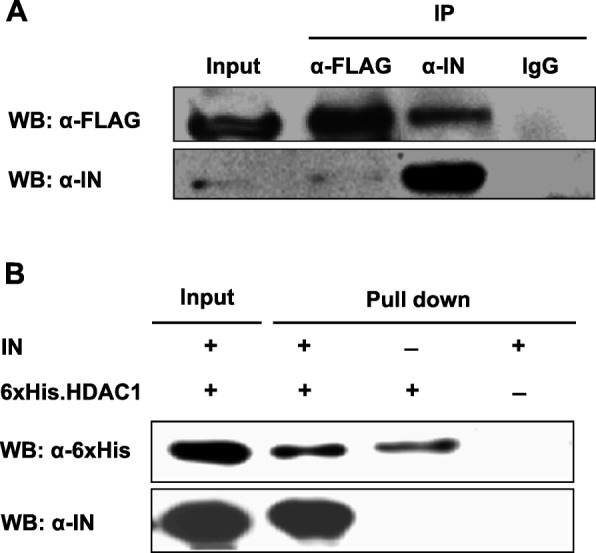
HDAC1 specifically interacts with HIV-1 integrase. **a** 293 T-IN^s^ala cells (stably expressing HIV-1 IN) were transfected with pFLAG-HDAC1. Cell lysates were then subjected to immunoprecipitation with anti-IN (α-IN), anti-FLAG (α-FLAG) or control IgG antibodies. Immunoprecipitates and total cell extracts (input) were analyzed by Western blot with anti-IN or anti-FLAG antibodies. **b** Purified IN was incubated with purified 6xHis-tagged HDAC1 or with Ni-NTA beads alone (negative control). Bound proteins and input were analyzed by Western blot using anti-6xHis or anti-IN antibodies

We next investigated the interaction of HDAC1 with the HIV-1 integrase by co-immunoprecipitation. Lysates from 293 T-IN^s^ala cells transfected with the pFLAG-HDAC1 expression vector were subjected to immunoprecipitation using anti-FLAG, anti-IN or control rabbit IgG antibodies. Immunoprecipitates were then analyzed with anti-FLAG and anti-IN antibodies. As shown in Fig. 2a, FLAG-HDAC1 co-immunoprecipitated with the integrase. The absence of FLAG-HDAC1 from control IgG immunoprecipitates indicated that this protein specifically interacted with the HIV-1 integrase. The interaction was also confirmed when extracts were immunoprecipitated with the anti-FLAG antibodies, despite a weak integrase signal observed upon Western blot analyses (due to poor abundance of the integrase protein in expressing cells). We further sought to validate this interaction by carrying out a pull-down assay using purified 6xHis-tagged HDAC1 and purified integrase. In this assay, integrase was detected following 6xHis.HDAC1 pull-down demonstrating a direct interaction between the two proteins (Fig. 2b).

### Transient knockdown of HDAC1 inhibits HIV-1 replication

**Fig. 3:**
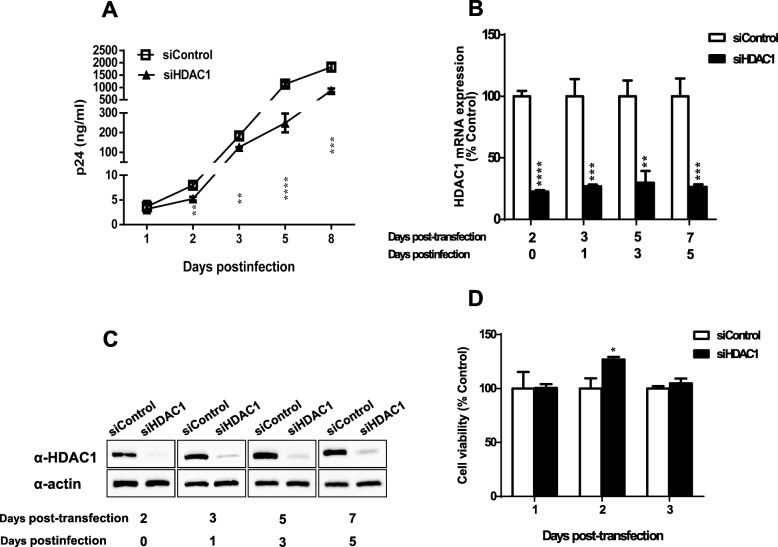
HDAC1 knockdown inhibits HIV-1 replication. **a** HeLa-CD4-LTR-β-gal cells were transfected with 15 nM siHDAC1 (Hs_HDAC1_5) or control siRNA (GFP-22 siRNA) and, 48 h after transfection, were infected with NL4–3 HIV-1 virions (10 ng p24 per 8 × 10^4^ cells). Culture supernatants were harvested at different time points post-infection and viral levels were measured through p24 ELISA assay. **b**, **c** siRNA-transfected HeLa-CD4-LTR-β-gal cells were harvested at indicated time points and analyzed for endogenous HDAC1 expression by quantitative RT-PCR (B) and Western blot (**c**). **d** Cell viability was determined at day 1, 2 and 3 after siRNA transfection, using the MTS/PMS assay. Results are expressed as percentage of the data obtained with control siRNA-transfected cells. Data in A, B, and D represent means ± SD of triplicates from a single experiment (*n* = 3). Results are representative of two independent experiments and were analysed by the Student t test (*, p < 0.05, **, p < 0.01 ***, p < 0.001, ****, p < 0.0001)

To study the impact of HDAC1 on HIV-1 replication, HDAC1-specific siRNA was tested in HeLa-CD4 cells in the context of multiple rounds of infection. HeLa-CD4 cells transfected with HDAC1 siRNA (Hs_HDAC1_5) or a non-related control siRNA (GFP-22 siRNA) were infected with HIV-1 NL4–3 and replication was monitored for 8 days by quantification of the p24 viral protein in cell supernatants at day 1, 2, 3, 5 and 8 post-infection (pi). The results showed a significant inhibition of HIV-1 replication in cells silenced for HDAC1 expression compared to control cells (Fig. 3a). Reduced replication was particularly prominent on day 5 pi where HDAC1 knockdown led to a 4.5-fold inhibition of HIV-1 replication compared to control.

The efficiency of transfected siRNAs was determined in parallel. As assessed by real-time quantitative PCR and Western blot analyses, a significant and steady reduction (70–80%) of HDAC1 expression levels was observed up to day 7 post-transfection (day 5 pi) with a maximum knockdown at day 2 (Fig. 3b, c). Cell viability assay also revealed no cytotoxicity (Fig. 3d). A similar reduction in HIV-1 replication was demonstrated with a different HDAC1-specific siRNA (Hs_HDAC1_6, data not shown).

**Fig. 4:**
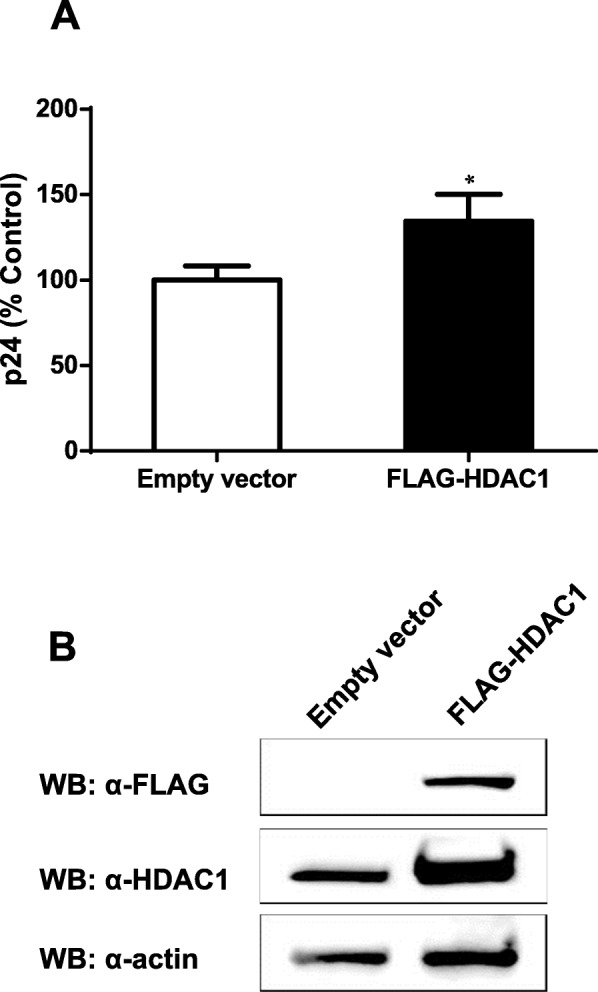
HDAC1 overexpression increases HIV-1 replication. **a** HeLa-CD4-LTR-β-gal cells were transfected with the pFLAG-HDAC1 expression vector or the pBact-FLAG empty vector (375 ng) and 48 h later infected with HIV-1 NL4–3 virions (10 ng p24 per 8 × 10^4^ cells). Supernatants were harvested at 48 h post-infection and measured for virus levels through p24 ELISA assay. Results are expressed as percentage of the control and represent means ± SD of triplicates from a single experiment (n = 3). Data are representative of two independent experiments and were analysed by the Student t test (*, *p < 0.05).* **b** Total cell lysates (48 h post-infection) was analyzed by Western blot with anti-FLAG, anti-HDAC1 and anti-β-actin antibodies

To further demonstrate the specific impact of HDAC1 on HIV-1 replication, we tested if its overexpression might lead to a significant change in HIV-1 replication. For this purpose, HeLa-CD4 cells were transfected with the p-FLAG-HDAC1 expression vector or with the empty vector (mock) and then infected with NL4–3 viruses. As shown in Fig. 4a, overexpression of HDAC1 in HeLa-CD4 cells induced a slight but significant increase in virus production compared to mock-transfected cells. This slight increase might be consequential to the limited overexpression of HDAC1 observed in these transfected cells, as denoted by Western blot analysis (Fig. 4b). Densitometry analyses with ImageJ indeed revealed a two-fold increase in HDAC1 levels in overexpressing cells following normalisation with the actin signals.

The above results thereby demonstrated that HDAC1 facilitates HIV-1 replication.

### HDAC1 knockdown affects HIV-1 replication at a preintegration step

**Fig. 5:**
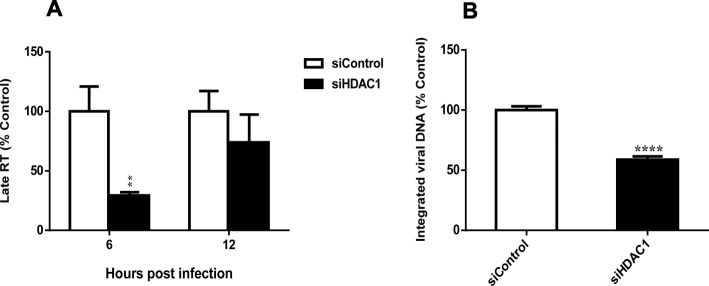
HDAC1 silencing decreases HIV-1 late reverse transcription. HeLa-CD4-LTR-β-gal cells were transfected with 15 nM siHDAC1 or control siRNA and infected with DNase I-treated HIV-1 NL4–3 virions (10 ng p24 per 8 × 10^4^ cells) 48 h after transfection. At different time point post-infection, cells were harvested and DNA was extracted. Late RT transcripts (6–12 h) (**a**) and integrated viral DNA (48 h) (**b**) were quantified by real-time PCR, as described in Materials and Methods. Results are expressed as percentage of the control (siControl-transfected cells are set as 100%). Data represent means ± SD of triplicates from a single experiment (n = 3) and were analysed by the Student t test (**, p < 0.01, ****, p < 0.0001). Data are representative of two independent experiments

Since we demonstrated that HDAC1 interacts with the HIV-1 integrase and regulates HIV-1 replication, we next investigated its impact on viral DNA integration. Integrated viral DNA and late RT products were thus quantified in siHDAC1- and siControl-transfected HeLa-CD4 cells infected with HIV-1 virions (Fig. 5). Levels of late RT species were significantly decreased in the HDAC1-knockdown cells compared to control cells at 6 h pi but were not significantly different at 12 h and later time point (24 h) (Fig. 5a; data not shown). The transient nature of the reduced late RT is likely representative of more efficient RT activity in HDAC1-expressing cells (vs. silenced cells), while the non-significant difference seen in later time points might be represent the remaining completed late RT products of non-silenced cells, which are then comparable to levels of late RT products, successfully but less efficiently completed in silenced cells. As predicted, HDAC1 knockdown further reduced integrated viral DNA levels by more than 40% (Fig. 5b). Similar results were obtained in HeLa-CD4 cells silenced for HDAC1 expression and infected with luciferase-expressing pseudotyped viruses (data not shown). Since decrease in integrated viral DNA levels is likely the result of decreased late RT, our results strongly suggest that the replication of HIV-1 in HDAC1-silenced cells is at least partly affected before integration, possibly at the reverse transcription step.

### HDAC1 modulates HIV-1 gene expression

**Fig. 6:**
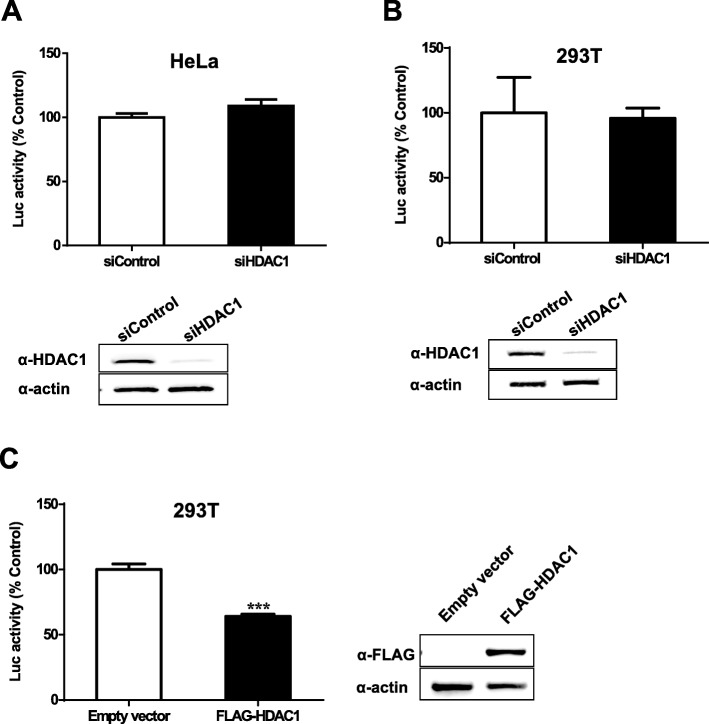
HDAC1 overexpression affects HIV-1 expression. **a**, **b** HeLa-CD4-LTR-β-gal (**a**) or 293 T (**b**) cells were transfected with 20 nM siHDAC1 (Hs_HDAC1_6) or control siRNA (GFP-22 siRNA) and, after 48 h, transfected with pNL4–3.Luc.R-E- (1.6 μg/10^5^ cells). HDAC1 knockdown was determined by Western blot analysis using anti-HDAC1 and anti-β-actin antibodies (lower panels). Luciferase activity was measured in cell lysates 24 h after proviral DNA transfection and normalized for protein levels. **c** 293 T cells were transfected with pFLAG-HDAC1 or the pBact-FLAG empty vector (375 ng per 10^5^ cells) and 48 h later, transfected with pNL4–3.Luc.R-E-. Luciferase activity was measured in cell lysates 24 h after proviral DNA transfection and normalized for protein levels. Cell lysates were analyzed by Western blot using anti-FLAG and anti-β-actin antibodies (right panel). Results derived from the measurement of luciferase activities were calculated as means ± SD of six measurements from a single experiment (*n* = 6) and are representative of two to four independent experiments. Results were analysed by the Student t test (***, p < 0.001)

We next analyzed the effect of HDAC1 on viral expression from proviral DNA. HeLa-CD4 or 293 T cells were transfected with siHDAC1 or siControl and further transfected after 48 h with pNL4–3.Luc.R-E- to bypass the integration step. HDAC1 knockdown was assessed by Western blot analysis as shown in Fig. 6 (a, b). Luciferase activity showed no significant difference between siHDAC1- and siControl-transfected cells (Fig. 6a, b, upper panels). These results thereby revealed that HDAC1 silencing does not affect HIV-1 expression in the context of the proviral DNA. On the other hand, we tested the effect of HDAC1 overexpression in 293 T cells and found that it decreased HIV-1 gene expression (Fig. 6c).

Our results thus showed that, while HDAC1 knockdown does not affect HIV-1 expression, its overexpression decreases proviral expression.

### Transient knockdown of HDAC1 in HIV-1 producer cells differently impacts replication in infected cells

We subsequently examined the effect of HDAC1 knockdown in HIV-1 producer cells on the infectivity of resulting virions. 293 T cells were first transfected with either siHDAC1 or siControl and subsequently co-transfected 48 h later with pNL4–3.Luc.R-E- clone and a VSV-G expression vector. Supernatants were harvested, normalized for p24 content and used to infect HeLa-CD4, Jurkat or human primary CD4+ T cells. In parallel, wild-type HIV-1 virions were produced in 293 T cells transfected with siHDAC1 or siControl and used to infect LuSIV cells.

**Fig. 7:**
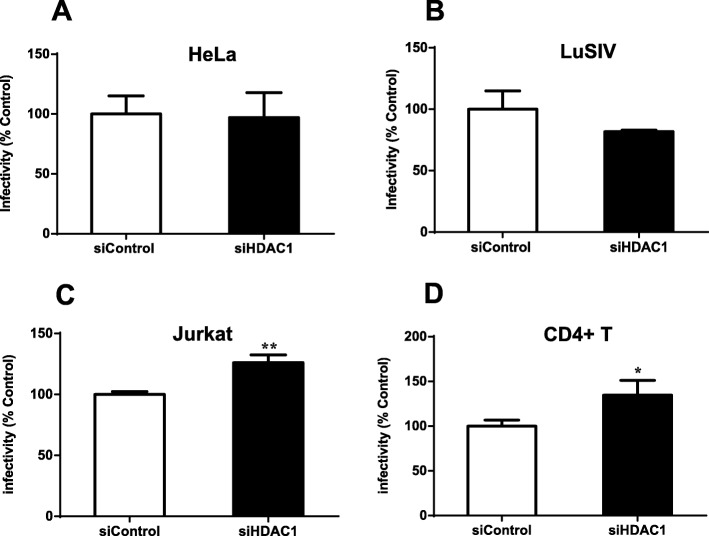
Knockdown of HDAC1 expression in HIV-1 producer cells affects virion infectivity in a cell type-dependent manner. 293 T cells were transfected with 20 nM siHDAC1 (Hs_HDAC1_6) or control siRNA (GFP-22 siRNA). At 48 h post-transfection, cells were transfected with pNL4–3 or co-transfected with pNL4–3.Luc.R-E- and pCMV-VSV-G vectors to produce wild-type or pseudotyped virions, respectively. Culture supernatants were harvested at 48 h after proviral DNA transfection and measured for virus production by p24 ELISA. Equal amounts of pseudotyped NL4–3.Luc.R-E- virions (p24 levels) were used to infect HeLa-CD4-LTR-β-gal (30 ng p24 per 10^5^ cells) (**a**), Jurkat (30 ng p24 per 10^6^ cells) (**c**) or primary CD4+ T cells (30 ng p24 per 10^6^ cells) (**d**). **b** LuSIV indicator cells were infected with NL4–3 virions (30 ng p24 per 10^6^ cells). Infectivity was measured in terms of luciferase activity and determined at 48 h post-infection. Results were calculated as a percentage of the control and represent means ± SD of six measurements from a single experiment (n = 6). Results are representative of two to three independent experiments and were analysed by the Student t test (*, *p* < 0.05, **, *p* < 0.01)

In HeLa-CD4 and LuSIV cells, infectivity of virions produced from siHDAC1 or siControl-transfected cells was comparable (Fig. 7a, b). However, virions produced from siHDAC1-transfected cells exhibited a slight albeit significant increase in infectivity in both Jurkat (Fig. 7c) and human primary CD4+ T cells (Fig. 7d), when compared to viruses produced from siControl-transfected cells. Our results suggest that HDAC1 knockdown in HIV-1 producer cells differently impacts virus infectivity, depending on the target cells.

## Discussion

In the present report, we provide evidence for the contribution of HDAC1 to HIV-1 replication. We demonstrate that it interacts with HIV-1 integrase and affects HIV-1 replication during early and late stages, as evidenced by siRNA-mediated knockdown and overexpression experiments.

HDAC1 belongs to the histone deacetylase family (HDACs) that is subdivided into four distinct classes (class I-IV). Class I HDACs include HDAC1, 2, 3 and 8 [42]. HDACs regulate transcription by acetylating the γ-amino groups of lysine residues of histone tails. As histone deacetylation is associated with inactive heterochromatin [43], the function of HDACs in HIV-1 replication has been mainly linked to viral gene expression and is at the basis of the use of specific inhibitors to reactivate HIV-1 latently infected cells [24].

Our results thus present a new mode, by which HDAC1 could contribute in regulating HIV-1 replication. Indeed, during the early events of HIV-1 replication, HDAC1 appears to be required before integration, likely at the reverse transcription step. We found that HDAC1 knockdown in HIV-1-infected cells decreased levels of late reverse transcripts early in infection. In line with a role of HDAC1 in reverse transcription, Sorin and co-workers have previously shown that the expression of a functionally inactive mutant of HDAC1 (HDAC1^H141A^) in HIV-1-producing cells led to an impairment of virus infectivity, due to a decrease in early and late reverse transcripts in infected cells [12]. Furthermore, the authors reported that HDAC1 was incorporated in HIV-1 virions in an integrase-dependent manner as part of the Sin3a-HDAC1 complex. A subsequent report has however showed that HDAC1 knockdown in infected cells increased HIV-1 integration [13]. The authors proposed that HDAC1 is recruited by KAP1, a protein belonging to the antiviral TRIM family, to deacetylate the HIV-1 integrase, leading to a decrease in viral integration. Acetylation of the integrase carboxy-terminal domain (CTD) by p300 and GCN5 histone acetyl transferases has been well demonstrated [44–46]. However, the requirement of this modification for integrase function and virus replication remains controversial [45]. Indeed, mutational analysis of integrase lysine residues (K264, K266, K273), commonly acetylated by p300 and GCN5, revealed that the loss of the acetylation potential of HIV-1 integrase did not impair virus replication [45, 47].

Several studies have demonstrated that HIV-1 integrase interacts with reverse transcriptase and that integrase mutations affect reverse transcription [2–4, 48–50]. Furthermore, two proteins, the integrase interactor protein 1 (INI1) and Gemin2, have been reported to interact with HIV-1 integrase and to affect reverse transcription efficiency [51–54]. In addition to histones, HDAC enzymes can modify a variety of non-histone proteins and regulate diverse processes including protein-protein and protein–DNA interactions, enzyme activity, protein stability, and subcellular localization [55, 56].

Our results also suggest that HDAC1 is involved in late events of HIV-1 replication. When the integration step was bypassed by transfection of proviral DNA constructs, we found that HDAC1 overexpression reduced luciferase activity, indicating that HDAC1 contributes to HIV-1 provirus repression. In contrast, HDAC1 siRNA in HeLa or 293 T cells did not affect HIV-1 LTR-driven expression of luciferase. Our results agree with an earlier report that demonstrated that the siRNA-mediated knockdown of HDAC1 in 293 T cells did not affect HIV-1 virion production [12]. On the other hand, the expression of a catalytically inactive dominant negative mutant of HDAC1 (HDAC1^H141A^) was shown to increase virion production [12]. Interestingly, HDAC1 and HDAC2 can function as homo- or heterodimers and the HDAC1^H141A^ mutant inhibits the activity of both proteins [57]. Thus, our results along with the findings reported above might suggest that both HDAC1 and HDAC2 are involved in the regulation of HIV-1 gene expression and that, in HDAC1 knockdown conditions, HDAC2 could persist in repressing HIV-1 gene expression. It has already been reported that HDAC inhibitors can induce viral expression in latently-infected cell line models and in resting CD4+ T cells obtained from aviremic HIV-1-positive patients [20, 21, 23]. Furthermore, HDAC1, HDAC2 and HDAC3 are known to be recruited to the HIV-1 LTR in latently-infected cell lines [14–19]. However, using targeted HDAC inhibition by specific siRNAs in HeLa cells, Keedy and co-workers observed that, in contrast to HDAC2 and HDAC3, HDAC1 knockdown did not increase HIV-1 LTR expression [58]. Furthermore, data from Barton and colleagues showed that HDAC1 depletion did not significantly induce HIV-1 expression from the HIV-1 promoter in a latency cell line model, as opposed to HDAC3 depletion alone or in combination with HDAC2 depletion [22]. These findings are consistent with our results and further indicate that the loss of HDAC1 may be compensated by other HDACs.

Our data further reveal that HDAC1 knockdown in HIV-1-producing cells differently affects virion infectivity, depending upon the targeted cell. HDAC1 depletion in producer cells has no effect on virion infectivity in HeLa or LuSIV cells. However, upon HDAC1 depletion in virion producer cells, virus infectivity slightly increased in Jurkat and human primary CD4+ T cells. This is in contrast with the results from Sorin and co-workers showing that the expression of the inactive HDAC1^H141A^ in producer cells led to a significant decrease in the infectivity of the virions in an osteosarcoma cell line [12]. Our results and those reported by Sorin et al. suggest that the effect of HDAC1 on HIV-1 infectivity depends on the cellular context. It will be interesting to pursue studies in each of these cell targets and further extend our analyses to HIV-1 latency models, such as J-Lat cells lines.

Overall, our data support that HDAC1 interacts with HIV-1 integrase and exerts a complex role in virus replication. Based on previous results and our own, we suggest that HDAC1 in target cells importantly acts at a late step of reverse transcription by positively modulating the reverse transcription process through a complex involving multiple cellular and viral factors such as the integrase, INI1 and Gemin2. In later step of the viral cycle, and, as extensively reported, HDAC1 acts as an inhibitor of viral expression in the nucleus. We further propose that HDAC2 and HDAC3 are more efficient than HDAC1 in regulating viral expression, although high expression levels of HDAC1 can further participate in this transcriptional repression. Additional data suggest that HDAC1 could influence virion infectivity (possibly through its incorporation in the particle itself) and might further contribute in early late step of viral infection, although this would likely depend on the cell type and possibly their HDAC1 levels. Further experiments are underway to address this model.

HDAC inhibitors are under investigation as part of a “shock-and-kill” strategy, which is based on the reactivation of HIV-1 expression in latently-infected cells with concomitant highly active therapies [23, 59]. However, it has been demonstrated that the HDAC inhibitor vorinostat (SAHA) promoted HIV-1 infection in CD4 + T cells, by enhancing the efficiency of post-entry events, including reverse transcription, nuclear import, and integration. Similarly, specific inhibition of HDAC6 by tubacin increased infection of CD4 + T cells by HIV-1 [60]. In this context and based on our results showing the complex role of HDAC1 in HIV-1 replication, these conflicting results strongly underscore the need to address the action of the different HDAC classes on early and late steps of HIV-1 replication in order to target the appropriate HDAC proteins and optimize active therapies.

## Conclusions

Using an integrase-LTR complex as a bait, we have demonstrated that HDAC1 is an interacting partner of the HIV-1 integrase. Our results further suggest that the implication of HDAC1 occurred at the preintegration step, although a role in HIV-1 replication at the post-integration step is also presented. Hence, HDAC1 through its association with the HIV-1 integrase, is a likely important modulator of HIV-1 replication. In light of the potential use of HDAC inhibitors as part of a “shock-and-kill” treatment, our study further underscores the importance of refining our comprehension of the role played by HDAC family members in the replication cycle of HIV-1.

## Supplementary information

**Additional file 1: Figure S1.** Complex formation between the HIV-1 LTR bait, integrase and LEDGF. (A) Biotinylated DNA fragments corresponding to the U3-LTR of HIV-1 was immobilized on streptavidin-coupled magnetic beads. Nuclear extracts from 293 T cells transfected with pCEP-IN^s^ala-Flag were incubated with the beads (coupled or not to the bait) and washed several times in cold lysis buffer. Bead and wash samples were boiled in Laemmli buffer for elution of the protein and resolved on a 10% SDS-PAGE followed by Western blot analyses with anti-FLAG antibodies. Total extracts (input) were similarly analyzed. (B) Nuclear extracts from 293 T cells transfected with CEP-IN^s^ala-Flag (vs. empty vector) and pcDNA3.1(+)-LEDGF-HA were incubated with HIV-1 U3 bait-coupled magnetic beads (vs. empty beads). After several washes, complexes were eluted from the beads in Laemmli buffer. Resulting eluted samples and bead-lysate complexes before wash were analyzed by Western blot with anti-HA antibodies.

## Data Availability

All data generated or analyzed during this study are included in this published article [and its supplementary information files].

## Abbreviations

CTD: carboxy-terminal domain
DMEM: Dulbecco’s Modified Eagle Medium
EMD: emerin
FBS: fetal bovine serum
FEN1: flap structure-specific endonuclease 1
HDAC1: histone deacetylase I
HIV-1: human immunodeficiency virus type 1
HMGB-1, HMGB-2: HMG-Box proteins
HSP60: heat shock protein 60
IN: integrase
INI1: integrase interactor protein 1
*LC-MS/MS*: liquid chromatography-tandem mass spectrometry
LEDGF/p75: lens epithelium-derived growth factor/transcription co-activator p75
LTR: long terminal repeat
MTS: tetrazolium salt [3-(4,5-dimethylthiazol-2-yl)-5-(3-carboxymethoxyphenyl)-2-(4-sulfophenyl)-2H-tetrazolium
PARP1: poly (ADP-ribose) polymerase 1
PBMCs: peripheral blood mononuclear cells
PEI: polyethylenimine
pi: post-infection
PIC: preintegration complex
PMS: phenazine methosulfate
XRCC5: X-ray repair cross complementing 5

## References

[1] Briones MS, Dobard CW, Chow SA (2010). Role of human immunodeficiency virus type 1 integrase in uncoating of the viral core. J Virol.

[2] Hehl EA, Joshi P, Kalpana GV, Prasad VR (2004). Interaction between human immunodeficiency virus type 1 reverse transcriptase and integrase proteins. J Virol.

[3] Zhu K, Dobard C, Chow SA (2004). Requirement for integrase during reverse transcription of human immunodeficiency virus type 1 and the effect of cysteine mutations of integrase on its interactions with reverse transcriptase. J Virol.

[4] Wilkinson TA, Januszyk K, Phillips ML, Tekeste SS, Zhang M, Miller JT (2009). Identifying and characterizing a functional HIV-1 reverse transcriptase-binding site on integrase. J Biol Chem.

[5] Mohammed KD, Topper MB, Muesing MA (2011). Sequential deletion of the integrase (gag-pol) carboxyl terminus reveals distinct phenotypic classes of defective HIV-1. J Virol.

[6] Ikeda T, Nishitsuji H, Zhou X, Nara N, Ohashi T, Kannagi M (2004). Evaluation of the functional involvement of human immunodeficiency virus type 1 integrase in nuclear import of viral cDNA during acute infection. J Virol.

[7] Jayappa KD, Ao Z, Yang M, Wang J, Yao X (2011). Identification of critical motifs within HIV-1 integrase required for importin alpha3 interaction and viral cDNA nuclear import. J Mol Biol.

[8] Masuda T, Planelles V, Krogstad P, Chen IS (1995). Genetic analysis of human immunodeficiency virus type 1 integrase and the U3 att site: unusual phenotype of mutants in the zinc finger-like domain. J Virol.

[9] Takahata T, Takeda E, Tobiume M, Tokunaga K, Yokoyama M, Huang YL, et al. Critical Contribution of Tyr15 in the HIV-1 Integrase (IN) in Facilitating IN Assembly and Nonenzymatic Function through the IN Precursor Form with Reverse Transcriptase. Journal of virology. 2017;91(1). 10.1128/JVI.02003-16 PMC5165222 27795445

[10] Vandegraaff N, Engelman A (2007). Molecular mechanisms of HIV integration and therapeutic intervention. Expert Rev Mol Med.

[11] Krishnan L, Engelman A (2012). Retroviral integrase proteins and HIV-1 DNA integration. J Biol Chem.

[12] Sorin M, Cano J, Das S, Mathew S, Wu X, Davies KP (2009). Recruitment of a SAP18-HDAC1 complex into HIV-1 virions and its requirement for viral replication. PLoS Pathog.

[13] Allouch A, Di Primio C, Alpi E, Lusic M, Arosio D, Giacca M (2011). The TRIM family protein KAP1 inhibits HIV-1 integration. Cell Host Microbe.

[14] Coull JJ, Romerio F, Sun JM, Volker JL, Galvin KM, Davie JR (2000). The human factors YY1 and LSF repress the human immunodeficiency virus type 1 long terminal repeat via recruitment of histone deacetylase 1. J Virol.

[15] Imai K, Okamoto T (2006). Transcriptional repression of human immunodeficiency virus type 1 by AP-4. J Biol Chem.

[16] Williams SA, Chen LF, Kwon H, Ruiz-Jarabo CM, Verdin E, Greene WC (2006). NF-kappaB p50 promotes HIV latency through HDAC recruitment and repression of transcriptional initiation. EMBO J.

[17] Jiang G, Espeseth A, Hazuda DJ, Margolis DM (2007). C-Myc and Sp1 contribute to proviral latency by recruiting histone deacetylase 1 to the human immunodeficiency virus type 1 promoter. J Virol.

[18] Marban C, Suzanne S, Dequiedt F, de Walque S, Redel L, Van Lint C (2007). Recruitment of chromatin-modifying enzymes by CTIP2 promotes HIV-1 transcriptional silencing. EMBO J.

[19] Tyagi M, Karn J (2007). CBF-1 promotes transcriptional silencing during the establishment of HIV-1 latency. EMBO J.

[20] Ylisastigui L, Archin NM, Lehrman G, Bosch RJ, Margolis DM (2004). Coaxing HIV-1 from resting CD4 T cells: histone deacetylase inhibition allows latent viral expression. AIDS..

[21] Archin NM, Espeseth A, Parker D, Cheema M, Hazuda D, Margolis DM (2009). Expression of latent HIV induced by the potent HDAC inhibitor suberoylanilide hydroxamic acid. AIDS Res Hum Retrovir.

[22] Barton KM, Archin NM, Keedy KS, Espeseth AS, Zhang YL, Gale J (2014). Selective HDAC inhibition for the disruption of latent HIV-1 infection. PLoS One.

[23] Archin NM, Liberty AL, Kashuba AD, Choudhary SK, Kuruc JD, Crooks AM (2012). Administration of vorinostat disrupts HIV-1 latency in patients on antiretroviral therapy. Nature..

[24] Manson McManamy ME, Hakre S, Verdin EM, Margolis DM (2014). Therapy for latent HIV-1 infection: the role of histone deacetylase inhibitors. Antivir Chem Chemother.

[25] Cravchik A, Matus A (1993). A novel strategy for the immunological tagging of cDNA constructs. Gene..

[26] Adachi A, Gendelman HE, Koenig S, Folks T, Willey R, Rabson A (1986). Production of acquired immunodeficiency syndrome-associated retrovirus in human and nonhuman cells transfected with an infectious molecular clone. J Virol.

[27] He J, Choe S, Walker R, Di Marzio P, Morgan DO, Landau NR (1995). Human immunodeficiency virus type 1 viral protein R (Vpr) arrests cells in the G2 phase of the cell cycle by inhibiting p34cdc2 activity. J Virol.

[28] Chang LJ, Urlacher V, Iwakuma T, Cui Y, Zucali J (1999). Efficacy and safety analyses of a recombinant human immunodeficiency virus type 1 derived vector system. Gene Ther.

[29] Cherepanov P, Maertens G, Proost P, Devreese B, Van Beeumen J, Engelborghs Y (2003). HIV-1 integrase forms stable tetramers and associates with LEDGF/p75 protein in human cells. J Biol Chem.

[30] Grandgenett DP, Goodarzi G (1994). Folding of the multidomain human immunodeficiency virus type-I integrase. Protein science : a publication of the Protein Society.

[31] Cherepanov P, Pluymers W, Claeys A, Proost P, De Clercq E, Debyser Z (2000). High-level expression of active HIV-1 integrase from a synthetic gene in human cells. FASEB J.

[32] Kimpton J, Emerman M (1992). Detection of replication-competent and pseudotyped human immunodeficiency virus with a sensitive cell line on the basis of activation of an integrated beta-galactosidase gene. J Virol.

[33] Roos JW, Maughan MF, Liao Z, Hildreth JE, Clements JE (2000). LuSIV cells: a reporter cell line for the detection and quantitation of a single cycle of HIV and SIV replication. Virology..

[34] Cantin R, Fortin JF, Lamontagne G, Tremblay M (1997). The presence of host-derived HLA-DR1 on human immunodeficiency virus type 1 increases viral infectivity. J Virol.

[35] Bounou S, Leclerc JE, Tremblay MJ (2002). Presence of host ICAM-1 in laboratory and clinical strains of human immunodeficiency virus type 1 increases virus infectivity and CD4(+)-T-cell depletion in human lymphoid tissue, a major site of replication in vivo. J Virol.

[36] Nilsen BM, Haugan IR, Berg K, Olsen L, Brown PO, Helland DE (1996). Monoclonal antibodies against human immunodeficiency virus type 1 integrase: epitope mapping and differential effects on integrase activities in vitro. J Virol.

[37] Najyb O, Brissette L, Rassart E (2015). Apolipoprotein D internalization is a Basigin-dependent mechanism. J Biol Chem.

[38] Li M, Craigie R (2005). Processing of viral DNA ends channels the HIV-1 integration reaction to concerted integration. J Biol Chem.

[39] Suzuki Y, Misawa N, Sato C, Ebina H, Masuda T, Yamamoto N (2003). Quantitative analysis of human immunodeficiency virus type 1 DNA dynamics by real-time PCR: integration efficiency in stimulated and unstimulated peripheral blood mononuclear cells. Virus Genes.

[40] Levis C, Fortini D, Brygoo Y (1997). Transformation of Botrytis cinerea with the nitrate reductase gene (niaD) shows a high frequency of homologous recombination. Curr Genet.

[41] Balakrishnan M, Jonsson CB (1997). Functional identification of nucleotides conferring substrate specificity to retroviral integrase reactions. J Virol.

[42] Bolden JE, Peart MJ, Johnstone RW (2006). Anticancer activities of histone deacetylase inhibitors. Nat Rev Drug Discov.

[43] Yang XJ, Seto E (2007). HATs and HDACs: from structure, function and regulation to novel strategies for therapy and prevention. Oncogene..

[44] Cereseto A, Manganaro L, Gutierrez MI, Terreni M, Fittipaldi A, Lusic M (2005). Acetylation of HIV-1 integrase by p300 regulates viral integration. EMBO J.

[45] Topper M, Luo Y, Zhadina M, Mohammed K, Smith L, Muesing MA (2007). Posttranslational acetylation of the human immunodeficiency virus type 1 integrase carboxyl-terminal domain is dispensable for viral replication. J Virol.

[46] Terreni M, Valentini P, Liverani V, Gutierrez MI, Di Primio C, Di Fenza A (2010). GCN5-dependent acetylation of HIV-1 integrase enhances viral integration. Retrovirology..

[47] Lu R, Ghory HZ, Engelman A (2005). Genetic analyses of conserved residues in the carboxyl-terminal domain of human immunodeficiency virus type 1 integrase. J Virol.

[48] Engelman A, Englund G, Orenstein JM, Martin MA, Craigie R (1995). Multiple effects of mutations in human immunodeficiency virus type 1 integrase on viral replication. J Virol.

[49] Wu X, Liu H, Xiao H, Conway JA, Hehl E, Kalpana GV (1999). Human immunodeficiency virus type 1 integrase protein promotes reverse transcription through specific interactions with the nucleoprotein reverse transcription complex. J Virol.

[50] Dobard CW, Briones MS, Chow SA (2007). Molecular mechanisms by which human immunodeficiency virus type 1 integrase stimulates the early steps of reverse transcription. J Virol.

[51] Nishitsuji H, Hayashi T, Takahashi T, Miyano M, Kannagi M, Masuda T (2009). Augmentation of reverse transcription by integrase through an interaction with host factor, SIP1/Gemin2 is critical for HIV-1 infection. PLoS One.

[52] Turelli P, Doucas V, Craig E, Mangeat B, Klages N, Evans R (2001). Cytoplasmic recruitment of INI1 and PML on incoming HIV preintegration complexes: interference with early steps of viral replication. Mol Cell.

[53] Maroun M, Delelis O, Coadou G, Bader T, Segeral E, Mbemba G (2006). Inhibition of early steps of HIV-1 replication by SNF5/Ini1. J Biol Chem.

[54] Hamamoto S, Nishitsuji H, Amagasa T, Kannagi M, Masuda T (2006). Identification of a novel human immunodeficiency virus type 1 integrase interactor, Gemin2, that facilitates efficient viral cDNA synthesis in vivo. J Virol.

[55] Batta K, Das C, Gadad S, Shandilya J, Kundu TK (2007). Reversible acetylation of non histone proteins: role in cellular function and disease. Subcell Biochem.

[56] Arif M, Selvi BR, Kundu TK (2010). Lysine acetylation: the tale of a modification from transcription regulation to metabolism. Chembiochem : a European journal of chemical biology.

[57] Luo Y, Jian W, Stavreva D, Fu X, Hager G, Bungert J (2009). Trans-regulation of histone deacetylase activities through acetylation. J Biol Chem.

[58] Keedy KS, Archin NM, Gates AT, Espeseth A, Hazuda DJ, Margolis DM (2009). A limited group of class I histone deacetylases acts to repress human immunodeficiency virus type 1 expression. J Virol.

[59] Colin L, Van Lint C (2009). Molecular control of HIV-1 postintegration latency: implications for the development of new therapeutic strategies. Retrovirology..

[60] Lucera MB, Tilton CA, Mao H, Dobrowolski C, Tabler CO, Haqqani AA (2014). The histone deacetylase inhibitor vorinostat (SAHA) increases the susceptibility of uninfected CD4+ T cells to HIV by increasing the kinetics and efficiency of postentry viral events. J Virol.

