# Supplementary figures and images for: Histone deacetylase 1 interacts with HIV-1 Integrase and modulates viral replication
Source: Virol J. 2019 Nov 19;16:138. doi: 10.1186/s12985-019-1249-y (PMC6862858; doi:10.1186/s12985-019-1249-y)

**A**

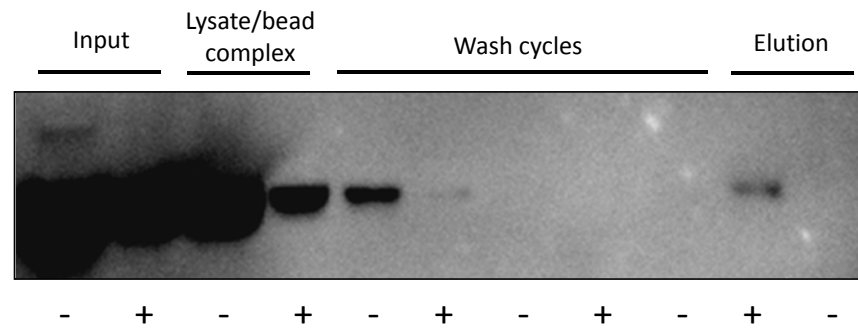

**B**

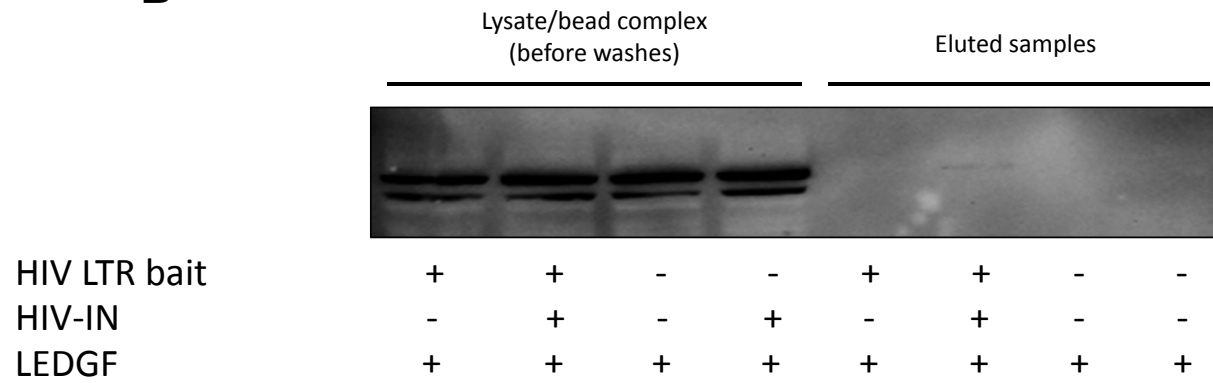

Supplement: Supplementary file 1 — Additional file 1: Figure S1. Complex formation between the HIV-1 LTR bait, integrase and LEDGF. (A) Biotinylated DNA fragments corresponding to the U3-LTR of HIV-1 was immobilized on streptavidin-coupled magnetic beads. Nuclear extracts from 293 T cells transfected with pCEP-INsala-Flag were incubated with the beads (coupled or not to the bait) and washed several times in cold lysis buffer. Bead and wash samples were boiled in Laemmli buffer for elution of the protein and resolved on a 10% SDS-PAGE followed by Western blot analyses with anti-FLAG antibodies. Total extracts (input) were similarly analyzed. (B) Nuclear extracts from 293 T cells transfected with CEP-INsala-Flag (vs. empty vector) and pcDNA3.1(+)-LEDGF-HA were incubated with HIV-1 U3 bait-coupled magnetic beads (vs. empty beads). After several washes, complexes were eluted from the beads in Laemmli buffer. Resulting eluted samples and bead-lysate complexes before wash were analyzed by Western blot with anti-HA antibodies. [file 12985_2019_1249_MOESM1_ESM.pdf]
